# Forced polarisation of microglia by IL-13 is modified by inflammatory and microenvironmental context

**DOI:** 10.1007/s00011-025-02089-2

**Published:** 2025-09-16

**Authors:** Emmanuelle D. Aiyegbusi, James P. Reynolds, Ross O’Carroll, Ruth Colbert, Christopher Carew, Dearbhaile Dooley

**Affiliations:** 1 School of Medicine, University College Dublin, Dublin, Ireland; 2 Conway Institute of Biomolecular and Biomedical Research, University College Dublin, Dublin, Ireland

**Keywords:** Microglia, IL-13, Polarisation, Spinal cord Injury, Neuroinflammation

## Abstract

**Background:**

Traumatic spinal cord injury (SCI) is a severe clinical challenge, often leading to long-term sensory, motor, and autonomic dysfunction. The SCI cascade involves a primary physical damage phase, followed by a secondary phase of inflammatory signalling driven by microglia and other infiltrating immune cells. Immunomodulatory therapies may help promote healing and restrict secondary damage. We have previously demonstrated that interleukin (IL)-13 delivery improves functional and histopathological recovery after SCI in murine models, primarily by polarising macrophages towards an alternatively activated pro-reparative M2-*like* phenotype and reducing axonal contacts. Although microglia respond robustly to IL-13 in vitro, polarisation of microglia in vivo is more difficult. To better understand what conditions may restrict microglial responses to IL-13 in vivo, we sought to examine the effect of cellular context or microenvironment on IL-13 efficacy in forcing microglia polarisation in vitro.

**Methods:**

BV2 and murine induced pluripotent stem cell (miPSC)-derived microglia were treated with IL-13 alone or in combination with lipopolysaccharide (LPS), acidic media, extracellular matrix components, high glutamate or high potassium concentrations. Following this phenotypic changes including morphology, gene/protein expression (TNFα, IL-1β, iNOS, Arg-1, CD206, F4-80) and cytokine release (TNFα) were measured using high-content screening, RT-qPCR, immunohistochemistry, and ELISA.

**Results:**

IL-13 leads to increased expression of the anti-inflammatory marker Arg-1 while lowering expression and secretion of the pro-inflammatory markers IL-1β, iNOS, and TNFα, and expression of the microglia activation marker F4-80, signifying effective polarisation of microglia. Concomitant administration of LPS with IL-13 reduces IL-13 polarisation efficacy in microglia. Forced polarisation of microglia is also compromised by high glutamate tone, acidosis, hyperkalemia, and extracellular fibronectin, suggesting microenvironmental contexts seen in neurotrauma directly act on microglia to limit polarisation potential.

**Conclusions:**

Our study demonstrates that the post-SCI environment dampens IL-13 efficacy on microglia. Taken together these data caution against simple immunomodulatory strategies and suggest that effective polarisation of microglia in vivo will require multimodal approaches.

## Introduction

Spinal cord injury (SCI) is a devastating condition leading to life-long difficulties among a relatively young patient cohort. In the central nervous system (CNS), primary neurotrauma entrains a secondary injury phase typified by inflammation and scar formation [1]. Although trauma-associated inflammation is critical in promoting wound corralling and limiting bystander damage [2], secondary inflammation in neurotrauma is associated with a chronic non-resolving pathology that limits restoration of function [3, 4]. Immunomodulatory strategies targeted at specific immune cell populations may hold promise in reducing chronic inflammation and promoting reparative processes [5–9]. However, following neurotrauma, extrinsic factors at the scar are likely to impose constraints on successful immunomodulation.

Microglia, as resident macrophages in CNS tissue, are the initial responders to neurotrauma, promoting monocyte infiltration and coordinating glial scar formation following trauma [10]. Pharmacological depletion of microglia through CSF1R inhibition has yielded mixed results, with reports that depletion can either improve functional recovery [11, 12] or lead to worsened outcomes following SCI [13, 14]. These inconsistencies may reflect the delayed changes in microglial phenotype from homeostatic to activated as the injury evolves [13, 15] and the emergence of mixed microglial phenotypes [10]. It also suggests that the gradual accumulation of SCI-associated extrinsic factors may constrain the range of possible phenotypes that microglia exhibit, inhibiting reparative processes. Such factors include those arising from fibrosis and gliosis, chronic inflammatory signals (arising from increased infiltration of immune cells as well as proliferation of activated microglia), acidosis, excitotoxicity, deposition of debris (including myelin), free radical production and loss of ionic homeostasis [1].

Recent studies have shown that microglia polarisation following SCI is influenced by the severity of the injury and the post-traumatic time window [16]. More severe injuries result in prolonged pro-inflammatory activation as indicated by high levels of inflammatory cytokines and chemokines including IL-1α, IL-1β, TNFα, RANTES, MCP-1, and IL-6 [16, 17]. Furthermore, the proliferative and phagocytic capacity by microglia is reduced. Molecular signals such as TNFα, IL-1β, IL-6, and glutamate promote M1-*like* phenotypes, while IL-4, IL-13, IL-10, and TGFβ support M2-*like* transitions [18, 19]. However, the SCI microenvironment often exhibit imbalances of cytokines, chemokines, neurotrophic factors, and ion channels. Neurons express pro-inflammatory cytokines quickly after injury, followed by microglia after 5 h. High levels of these cytokines enhance microglia activation thus promoting neuronal damage, demyelination, and blood-spinal cord barrier breakdown due to stimulation of neurotoxic genes i.e., COX2 and iNOS [20, 21]. The infiltration of immune cells from the periphery and neuronal cell death contributes to M1-*like* microglia polarisation [22]. Disrupted K^+^ channels can further increase demyelination and conduction failure, perpetuating injury signals that sustain M1-*like* microglial activation [20, 23]. Na^+^ and Ca^+^ ion channel dysfunction causes cellular oedema and acidosis indirectly promoting M1-*like* microglial activation via cellular stress. Post-SCI, there is iron accumulation which exacerbates oxidative stress and glutamate cytotoxicity which contribute to inflammatory microglia activation.

**Fig. 1:**
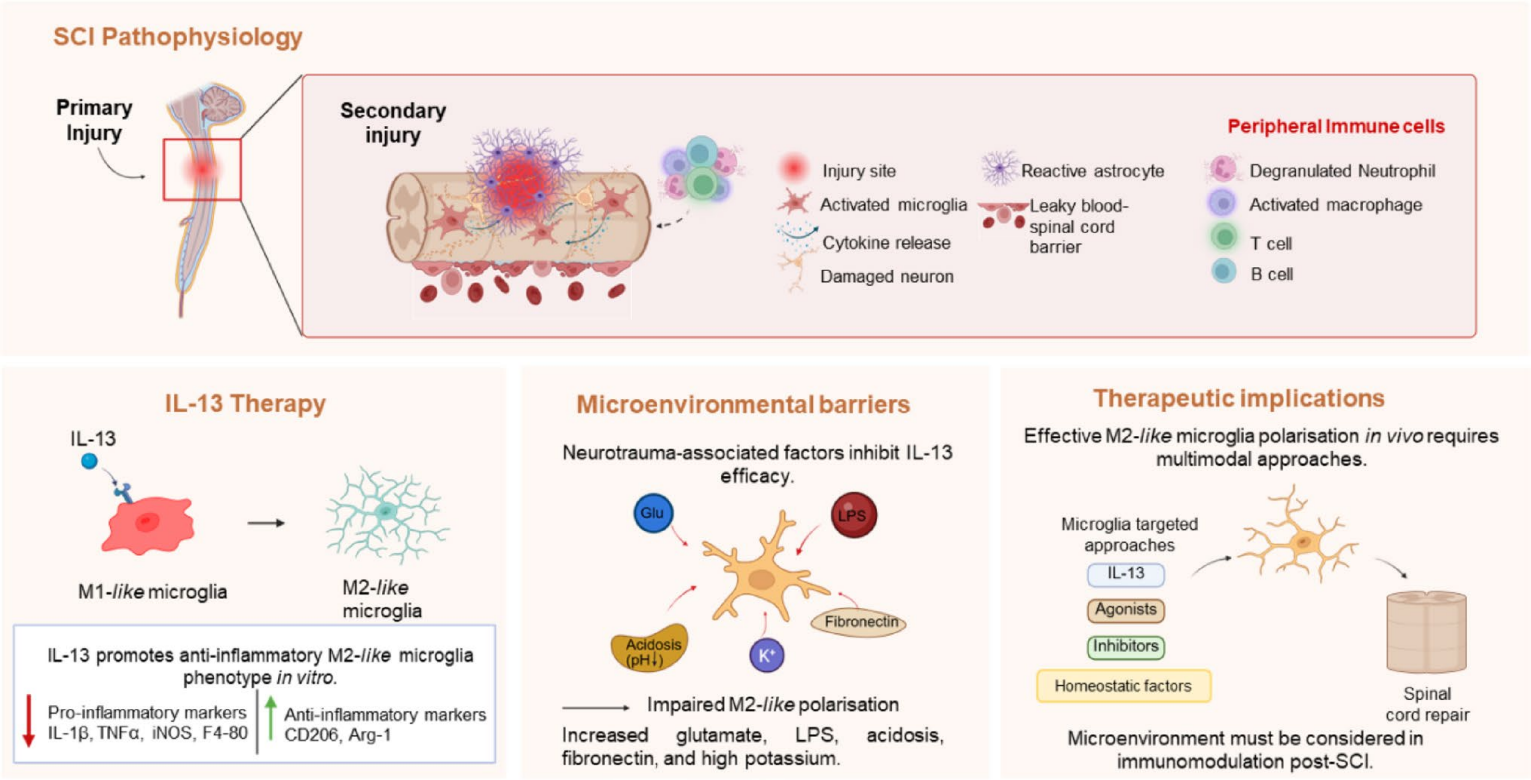
The first panel outlines mechanical disruption of spinal cord tissue and complex secondary injury phase, including glial scar formation, microglia activation, neuronal damage, and peripheral immune cell infiltration. The second panel illustrates IL-13 induced phenotypic shift in microglia from a pro-inflammatory M1-*like* profile to an anti-inflammatory M2-*like* phenotype, marked by elevated expression of CD206 and Arg-1. The third image identifies in vivo obstacles to IL-13 therapy, such as excess glutamate, LPS, acidic pH, elevated potassium, and fibronectin deposition, that hinder M2-*like* microglia polarisation. The fourth visual proposes a multifaceted approach combining IL-13 with agonists, homeostatic factors, and/or targeted inhibitors to promote M2-*like* microglia polarisation, reduce inflammation, support tissue repair, and improve recovery outcomes. Figure created using BioRender

We have previously demonstrated that interleukin-13 (IL-13) can successfully polarise monocyte-derived macrophages (MDMs) toward alternative activation states (referred to as M2-*like* phenotypes [24, 25]) leading to improved motor recovery following SCI [5, 26]. Although IL-13 can induce M2-*like* states in microglia that are associated with pro-reparative and anti-inflammatory functions [9], microglia were resistant to IL-13-mediated polarisation in SCI [5]. It is likely that extrinsic factors in the injured cord constrain microglial responses to IL-13, but a better understanding of which specific factors are responsible is needed to improve immunomodulatory approaches targeted at microglia. Here, we confirmed that cultured microglia originating from both immortalised cell lines and murine induced pluripotent stem cell (miPSCs) sources, reliably undergo M1-*like* and M2-*like* transitions that could be used to assay sensitivity to forced polarisation by IL-13. We investigated several parameters relevant to SCI conditions that we hypothesised could limit IL-13 polarisation. We noted that several such parameters lead to quiescent or mixed phenotypes in microglia with limited anti-inflammatory potential. These data caution against single-mode immunomodulation and suggest forced polarisation of microglia will require multimodal approaches that circumvent the constraints imposed by extrinsic factors arising during SCI (Fig. 1).

## Materials and methods

### Immortalized BV2 microglia cell culture

BV2 cells were generously gifted by Derek Costello, University College Dublin. BV2 cells were cultured in Dulbecco’s Modified Eagle’s Medium (DMEM; Corning, 15–013-CM) supplemented with fetal bovine serum (FBS 10%; Gibco, 10,270,106), Penicillin–Streptomycin (1%; Gibco, 15,070,063), and HEPES (15 mM; Sigma-Aldrich, H0887-100 mL) at 37 °C with 5% CO_2_ saturation. The cells were routinely passaged at 80–90% confluency using trypsin. All data were derived from passages 11 through 18. BV2 microglia were seeded on surface-treated plastic (6-well plate; Greiner, 657,160) or poly-D-lysine (0.1 mg/ml; Gibco, A3890401)-coated glass. Glass coverslips (13 mm; VWR, 631–0149) were coated with 100 mL of poly-D-lysine for 24 h at room temperature.

### Neural stem cell culture

Neural stem cells (NSCs) were isolated from day 14–16 mouse embryos of wildtype C57BL/6 embryos (male and female) following a previously described protocol [27] and cultured in Dulbecco’s Modified Eagle Medium (DMEM) and Ham’s F-12 Nutrient Mixture (DMEM/F12; Corning, 10–092-CV) supplemented with B27 (1X; Gibco, 17,504,044), Penicillin–Streptomycin (1%; Gibco, 15,070,063), and fungizone (0.2%; Gibco, 15,290,018). NSCs were passaged at a ratio of 1:3 every 7 days using accutase (Gibco, A1110501) for cell detachment. NSCs were seeded in fibronectin (10 mg/ml; Bio-Techne, 1030-FN-05 M) coated 96 well PhenoPlates (Revvity, 6,055,302) at a density of 31,250 cells/cm^2^ for 2 days prior to co-culture with miPSCs.

### Murine iPSC-derived microglia culture

Mouse embryonic fibroblasts (MEFs) and CX3CR1eGFP/ + CCR2RFP/ + miPSCs were gifted by Prof. Peter Ponsaerts, University of Antwerp, Belgium. Irradiated MEFs were cultured in DMEM (Corning, 10–013-CV) supplemented with FBS (10%; Gibco, 10,270,106), Penicillin–Streptomycin (1%; Gibco, 15,070,063), and Minimum essential medium, Non-Essential Amino Acids Solution (1X MEM-NEAA; Gibco, 11,140,035) at 37 °C with 5% CO_2_ saturation. Irradiated MEFs were seeded in surface treated 6-well plates (Greiner, 657,160). After 24 h, miPSCs were seeded on top of the irradiated MEFs and cultured in Knock-out DMEM (KO-DMEM; Gibco 10,829,018) supplemented with Embryonic stem cell FBS (ESC-FBS 15%; Gibco 10,439,001), Penicillin–Streptomycin (1%; Gibco, 15,070,063), L-Glutamine (2 mM; Gibco, 25,030,024), MEM-NEAA (1X; Gibco, 11,140,035), 2-mercaptoethanol (0.24 mM; Gibco, 31,350,010), and Recombinant mouse Leukemia Inhibitory Factor (LIF 10^6^ U/ml; Sigma-Aldrich, ESG1106). After two days in culture, miPSCs were passaged 1:5 following detachment using 0.05% Trypsin–EDTA (Gibco, 25,300,054) and seeded onto a new layer of irradiated MEFs. Murine iPSCs were generated following the protocol previously described [27]. Briefly, 500,000 miPSCs were seeded per agarose-coated 10 cm petri dishes in microglia differentiation medium (MDM) consisting of Glasgow’s minimum essential medium (GMEM; Gibco, 21,710,025) supplemented with FBS (10%; Gibco, 10,270,106), Penicillin–Streptomycin (1%; Gibco, 15,070,063), MEM-NEAA (1X; Gibco, 11,140,035), Sodium pyruvate (1 mM; Gibco, 11,360,039), and 2-mercaptoethanol (0.1 mM; Gibco, 31,350,010). Embryoid bodies (EBs) formed were transferred to new agarose-coated dishes on day 4. On day 8, the EBs were transferred into gelatin-coated (0.1% in sterile dH_2_0 for 1 h; Sigma-Aldrich, G1393-20ML) T175 flasks in MDM supplemented with SCF (200 ng/ml; ImmunoTools, 12,343,327) and VEGF-A (10 ng/ml; ImmunoTools, 12,343,663). On day 11, the medium was changed to MDM supplemented with 15% (v/v) L-929 conditioned media, 20% (v/v) bEND5 conditioned media, IL-3 (2 ng/ml; ImmunoTools, 12,340,033), and GM-CSF (40 ng/ml; PeproTech, 315–03). On day 15, the media was changed back to the original MDM. From days 29–57, GFP⁺ floating cells called primordial microglia progenitors (PMPs) released by the EBs were harvested and cultured at a density of 31,250 cells/cm^2^ on a layer of NSCs for 7 days in MDM as described previously by Quarta [27] and Haenseler et al., [28] who demonstrated that this window supports microglial marker expression in a neuroectodermal setting. These NSC/miPSC microglia cultures were used for stimulation experiments, subsequent enzyme-linked immunosorbent assay (ELISA) and/or immunocytochemistry experiments.

### Cell treatments

To investigate inflammatory responses in microglia, BV2 cells were treated for 24 h with LPS (InvivoGen, tlrl-b5lps) and/or IL-13 (ImmunoTools, 12,340,137) at concentrations of 1, 10, 100 or 1000 ng/ml and 20, 100, or 500 ng/ml, respectively. Cells were starved in 0.5% FBS during treatment. For timed dual stimulation experiments, BV2 cells were pre-treated with LPS (100 ng/ml) for 24 h before being subsequently treated with either low serum media control, LPS (100 ng/ml), or IL-13 (20 or 100 ng/ml) for an additional period of 6, 24, or 48 h. To investigate inflammatory responses in miPSC-microglia, the NSC/miPSC microglia co-cultures were treated for 24 h with LPS (InvivoGen, tlrl-b5lps) and/or IL-13 (ImmunoTools, 12,340,137) at concentrations of 1, 10, or 100 ng/ml and 20, 100, or 500 ng/ml, respectively; treatments were prepared in MDM.

To determine how matrix proteins impact IL-13 response, BV2 cells were grown in 6-well plates coated with collagen IV (10 mg/ml; Bio-Techne, 3410–010-02) or fibronectin (10 mg/ml; Bio-Techne, 1030-FN-05 M). Plates were coated for 2 h and then washed three times with sterile 1X DPBS without Ca^2+^ and Mg^2+^ (Gibco, 14,190,144). To assess how hyperkalemia or excessive glutamate impacts IL-13 response in microglia, BV2 or miPSC-microglia cells were treated in media supplemented with either [K^+^]_e_ (Fisher Chemical, P/4240/60), 15 mM or 35 mM final concentration for BV2 cells and 10 mM or 20 mM final concentration for miPSC-microglia or glutamate (Sigma-Aldrich, G1626-100G), 2 mM or 20 mM final concentration. To assess the polarising ability of IL-13 on BV2 microglia under acidic conditions, BV2 growth media was adjusted from a pH of 7.3 to a pH of 6.7 and sterile filtered before use with 0.22 μm syringe filter (Merck, SLGP033RS).

When assessing the impact of matrix proteins, potassium, glutamate and pH on IL-13 response, BV2 cells were seeded at a density of 200,000 cells per well and left to proliferate for 24 h in growth media. Following this, the cells were starved with 2% serum-containing media for another 24 h before being treated with IL-13 (100 ng/ml, for 24 h), prepared using 2% FBS-containing media. The cells were then lysed with TRIzol for RT-qPCR analysis. PMPs were cultured at a density of 31,250 cells/cm^2^ on a layer of NSCs for 7 days in MDM. Following this, NSC/miPSC- microglia were treated for 24 h with IL-13 (100 ng/ml).

### Enzyme-linked immunosorbent assay

The concentration of TNFα in the cell supernatants were determined using ELISA according to the instructions provided by the manufacturer (Bio-Techne, DY410-05).The culture supernatant was collected from the wells of a 6 or 24 well plate that had been seeded with BV2 cells and treated with LPS and/or IL-13, or 96 well PhenoPlates seeded with NSC/miPSC-microglia treated with LPS and/or IL-13, [K^+^]_e_ (10 mM or 20 mM final concentration) or glutamate (2 mM or 20 mM final concentration). A 96 well plate was coated with the capture antibody diluted in plate coating buffer (800 ng/ml), sealed, and incubated overnight. Following the overnight incubation, the wells of the plate were aspirated and washed with wash buffer thrice. The wells were subsequently blocked with BSA (1% in PBS) for 1 h at room temperature. The aspiration and wash step were repeated. 100 ml of the supernatants was added to each well of the 96-well plate and incubated for 2 h at room temperature. The aspiration and wash step were repeated. The plate was subsequently incubated with detection antibody diluted in reagent diluent (37.5 ng/ml) for 2 h at room temperature. The aspiration and wash step were repeated. Following this, the plate was incubated with streptavidin–horseradish peroxidase (40-fold dilution in PBS with 1% BSA) for 20 min in the dark at room temperature. The final aspiration and wash step were repeated. Substrate reagent was added to the wells and incubated for 20 min at room temperature. Following this, sulfuric acid (2N) was added to stop the reaction. Immediately after, the optical density was measured at 450 nm and 570 nm a microplate reader (Molecular Devices, 89,212–396 (PLUS 384)). The readings at 570 nm were subtracted from the readings obtained at 450 nm prior to analysis and analyte concentration was determined in relation to known TNFα standards.

### RNA isolation and quantitative real-time PCR

Total RNA was extracted from BV2 microglia using TRIzol (Thermo Fisher Scientific, 15,596,018). Cells were gently washed using ice-cold 1X DPBS prior to lysis with TRIzol. Following lysis, RNA was isolated via phase separation with chloroform, precipitation with isopropanol, and after two washes with 75% ethanol, the RNA pellet was dissolved in RNase-free H_2_O. 2 μl of GlycoBlue (ThermoFisher, AM9516) was added as a co-precipitant. The concentration of RNA was measured using a Spectrophotometer (DeNovix, DeNovix DS-11). Sufficient sample purity was accepted at values of > 1.8 for the 260/280 ratio and > 2.0 for the 260/230 ratio. cDNA was synthesised from total RNA using SuperScript™ II Reverse Transcriptase (Thermo Fisher Scientific, 18,064,014). All quantitative reverse transcription polymerase chain reactions (qRT-PCR) were performed in 384-well plates on a QuantStudio™ 7 Flex Real-Time PCR System (Thermo Fisher Scientific, 4,485,701) using SYBR Green PCR Master Mix Reagent (Thermo Fisher Scientific, 4,364,344) with Quantstudio Real-Time PCR software. Peptidylprolyl Isomerase A (PPIA) was used to normalise expression levels. The 2^−ΔΔCT^ method was used to calculate relative changes in gene expression. The sequences used for the primers are shown in Table 1.

**Table 1:** Primers for RT-qPCR

Gene	Forward primer (5’-3’)	Reverse primer (5’-3’)
IL-1β	TGCCACCTTTTGACAGTGATG	ATGTGCTGCTGCGAGATTTG
TNFα	AGCCGATGGGTTGTACCTTG	ATAGCAAATCGGCTGACGGT
iNOS	CAGATCGAGCCCTGGAAGAC	GTGAAGCCATGACCTTTCGC
CD206	GTGGACGCTCTAAGTGCCAT	GAATCTGACACCCAGCGGAA
Arg-1	AGGGTTACGGCCGGTGGAGAG	CCTCAGTGCTGCAGGGCCTTT
PPIA	CGTCTCCTTCGAGCTGTTTG	CACCACCCTGGCACATGAAT

IL-1β: Interleukin 1 beta. TNFα: Tumour necrosis factor alpha. iNOS: inducible nitric oxide synthase. Arg-1: Arginase 1. PPIA: Peptidylprolyl Isomerase A

### Scratch wound assay

To assess infiltration of wound areas by microglia, BV2 cells were grown for 2 days in DMEM supplemented with (10%; Gibco, 10,270,106), Penicillin–Streptomycin (1%; Gibco, 15,070,063), and HEPES (15 mM; Sigma-Aldrich, H0887-100 mL), cultured at 37 °C with 5% CO_2_ saturation as above, and treated with either IL-13 or LPS. After 24 h, when the monolayer is typically ~ 90% confluent, a ~ 250 µm scratch was made. The cells were gently washed with DMEM and low-serum media containing treatments (LPS or IL-13) was then added. Infiltration was determined 24 h later, using image binarisation to determine the relative change in scar density as compared to untreated controls.

### Immunocytochemistry

To perform immunocytochemistry (ICC), cells were first washed in PBS on a shaker for 10 min at room temperature. The cells were then blocked for 30 min at room temperature using protein block (Abcam, ab64226) After blocking, the primary antibodies, Anti-MHCII (Abcam, ab180779), Anti-Iba1 (FujiFilm Wako, 019–19741), Anti-Arginase 1 (Abcam, ab91279) and Anti-F4/80 (BioRad, MCA497GA) were added at a dilution of 1:400 using an antibody diluent consisting of 1% protein block and 0.05% Triton-X in 1X PBS, and incubated with the cells overnight at 4 °C. Next, the cells were washed three times for 5 min each in 1X DPBS on a shaker in the dark. The secondary antibodies Goat anti-rabbit 568 (Invitrogen, A11011) and, Goat anti-rat 647 (Invitrogen, A21247) were added at a dilution of 1:400 and incubated with the cells for 2 h at room temperature in the dark. The cells are again washed three times for 5 min each in 1X PBS on a shaker in the dark. When performing high-content screening, BV2 cells were stained with a green cell mask (Invitrogen, C37608) for 30 min at room temperature, to label the cell membrane for morphological analysis, the cells were then washed with 1X PBS. To visualise the nuclei, the cells were counterstained with 300 nM Hoechst 33342 for 10 min at room temperature in the dark. The cells were washed thrice with 1X PBS and subsequently kept in PBS prior to imaging on an Olympus FV1000 microscope (using 405 nm, 488 nm and/or 559 nm laser-scanning confocal imaging) or an Opera Phenix High-Content Screening optical system (see below).

### Calcium imaging

To register live calcium activity in microglia, BV2 cells were grown in complete DMEM on poly-D-lysine-coated 35 mm glass confocal dishes for two days as above. Media was exchanged for low-serum (2%) complete DMEM and cells were treated with either LPS or IL-13. The cells were washed three times with warmed sterile PBS and then incubated with Fluo4-AM (Thermo Fisher Scientific, F14201) in Hanks’ balanced salt solution (HBSS) (Sigma-Aldrich, D8537) for 1 h, 37 °C. Cells were washed once and imaged at 37 °C in artificial cerebrospinal fluid (aCSF, in mM; NaCl, 125; KCl, 2.5; NaH2PO4, 1.25; NaHCO3, 11.9; CaCl2, MgSO4∙7H2O, 1.3; D-glucose, 18; HEPES, 10). Calcium imaging was performed using a Nikon eclipse TS100 brightfield microscope with a custom-fitted fluorescence microscopy module. A 470 nm LED (Thorlabs, M470L5) was mounted to the rear optical port and routed through a FITC-filter cube, consisting of a 475/35 nm bandpass (excitation) filter (Thorlabs, MF475-35), a 530/43 nm bandpass (emission) filter (Thorlabs, MF540-43), and a 498 nm longpass dichroic mirror (Thorlabs, MD498). Fluorescent images were acquired at 2 Hz using an Optika CP-6 digital camera (Optika Italy, 57,549) and a Nikon Plan Fluor 20X 0.45 NA objective. Calcium imaging performed here is as described in [29].

#### High-content screening

To examine the expression of anti-inflammatory markers by BV2 or NSC/miPSC-microglia cells in response to pro- and anti-inflammatory stimuli, immunofluorescence and high content screening were carried out using the Opera Phenix high-throughput confocal microscope (Revvity, HH14001000). Fluorescence intensity and cell morphology were analysed using the Harmony software (Harmony® high-content analysis software). For BV2 cell analysis, 96-well optical plate (Revvity, 6055302) was coated with 100 ml Poly-D-Lysine (0.1 mg/ml, Gibco, A3890401) for 4 h at room temperature. The plate was subsequently washed with PBS thrice. 10,000 BV2 cells were seeded per well and left to proliferate for 48 h. Following this, the cells were starved with 2% serum media for another 24 h. Subsequently, BV2 cells were treated with IL-13 (100 ng/ml), LPS (100 ng/ml), TGFβ1 (50 ng/ml; Biolegend, 763,104), TGFβ2 (50 ng/ml; Biolegend, 583,301), M-CSF (10 ng/ml; Peprotech, 315–02), and/or ovine cholesterol (Avanti Polar Lipids / Merck Millipore, 700000P). After 24 h of treatment, the cells were fixed with 2% paraformaldehyde (PFA) for 20 min. The cells were washed thrice with DPBS for 5 min before and after fixation. PMPs were developed on a layer of NSCs for 6 days, on day 7, the NSC/miPSC-microglia were treated with IL-13 (100 ng/ml), LPS (100 ng/ml), TGFβ1 (50 ng/ml), TGFβ2 (50 ng/ml), M-CSF (10 ng/ml), and/or ovine cholesterol. Alternatively, NSC/miPSC-microglia were treated with KCl (10 mM or 20 mM final concentration [K^+^]_e_) or glutamate (2 mM or 20 mM final concentration) to quantify the effects of microenvironmental factors on pro- or anti-inflammatory marker expression. Following 24 h of treatment, the cells were washed once with DPBS, fixed for 20 min using 2% PFA, and then washed thrice with DPBS.

#### Statistical analysis

A minimum number of three experimental replicates with at least two technical replicates was used in all experiments unless otherwise noted. For the statistical tests the following analyses of variance were used; Unpaired t-test, one-way ANOVA with Šídák’s or Dunnett’s multiple comparison tests; two-way ANOVA with Benjamini, Krieger and Yekutieli False Discovery Rate post-hoc test. Results were considered significant at *P* < 0.05. Statistical significance was denoted by *P < 0.05, **P < 0.01, ***P < 0.001, and ****P < 0.0001.

## Results

### IL-13 induces alternative activation of microglia

**Fig. 2:**
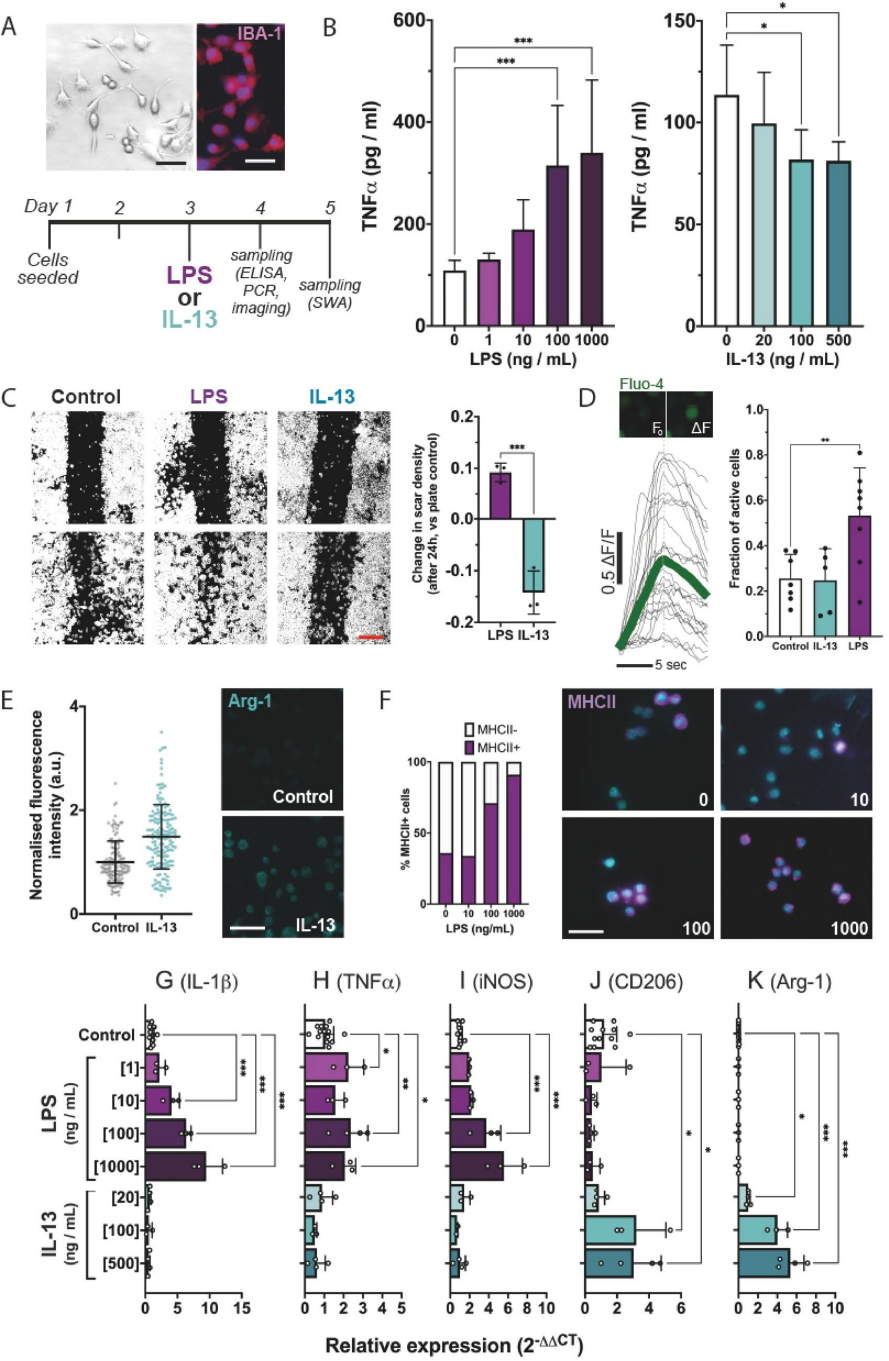
IL-13 induces alternative activation of microglia. **A** To investigate how the microglial response to IL-13 is distinct from classical inflammatory activation, BV2 cells were seeded in multi-well culture vessels and incubated for 2 days. On Day 3, cells were treated with LPS (1, 10, 100 or 1000 ng / mL) and/or IL-13 (20, 100 or 500 ng / mL) prior to sampling for ELISA, RT-qPCR or fluorescence imaging, 24 h later. SWA was initiated 24 h following treatment and assessed 24 h later. Scale bar, 20 μm. **B** TNFα secretion was measured using ELISA, with supernatant sampled at 24 h after treatment with either LPS or IL-13. Data shown as mean ± SD; n = 5–9 biological replicates from at least 3 independent cultures. **C** SWA was performed on confluent BV2 cell monolayers. A scratch of ~ 250 μm width was inflicted 24 h after treatment with either LPS (100 ng/mL) or IL-13 (100 ng/mL). Binary cell masks were used to determine the extent of re-infiltration of the original scratch wound 24 h later (while continuing LPS or IL-13 treatments). Scale bar, 200 μm. *Right*, the change in scar density, relative to matched-controls, was computed to directly compare LPS and IL-13 treatments. Aggregate data shown as mean ± SD; n = 3 biological replicates from independent cultures. **D** Fluo-4 AM loaded BV2 cells were imaged at 3 Hz following treatment for 24 h with either LPS (100 ng / mL) or IL-13 (100 ng / mL). The prevalence of Ca^2+^ transients (defined as an increase of > 0.1 DF/F_0_, elapsing > 3 s) occurring per cell per 120 secs was computed to compare between treatments. Data shown as mean ± SD; n = 5–8 biological replicates from at least 3 independent cultures. *Inset, upper left*, example of fluorescence change arising during a somatic calcium elevation. **E** Confocal imaging of microglia, immunostained for Arg-1, at 24 h following IL-13 treatment (100 ng/mL). Mean cell fluorescence intensity was plotted as mean ± SD; representative data from a single experiment. Scale bar, 50 μm. Statistical analysis performed using an unpaired t-test. **F** Epifluorescent imaging of microglia, immunostained for MHC-II, at 24 h following LPS treatment (10, 100 and 1000 ng/mL). Immunostaining was performed without permeabilisation, to limit fluorescence to membrane-bound MHCII only, and the proportion of MHC-II-positive cells was determined in order to compare treatments. Representative data from a single experiment. Scale bar, 50 μm. **G-K** Relative transcription of M1-*like* genes IL-1β (**G**), TNFα (**H**), and iNOS (**I**), and of M2-*like* genes CD206 (**J**) and Arg-1 (**K**) were measured through quantitative PCR, using PPIA as an endogenous reference gene. Cells were treated with either LPS or IL-13 at various doses, and RNA was sampled 24 h later. Data shown as mean ± SD; n = 3–13 biological replicates from at least 3 independent cultures. One-way ANOVA with Dunnett’s multiple comparisons test was applied to panels **B**, **D**, and **G–K**. Unpaired t-tests were used for panels **C** and **E**. Unless specified by pairwise annotations, * specifies differences versus control

IL-13 is a regulatory cytokine known to induce an alternative activation state in myeloid cells [30] via JAK / STAT signalling that leads to changes in intracellular signalling, inflammatory behaviour (including cytokine release) and injury response. First, to establish how IL-13-induced activation of microglia differs in comparison to the known pro-inflammatory stimulant LPS, BV2 cells were treated with either LPS or IL-13 (Fig. 2A). We assayed differences in cellular behaviours (including scratch wound response and intracellular calcium elevations) as well as expression and secretion of factors associated with classical (M1-*like*) and alternative (M2-*like*) activation.

IL-13 induced distinct states in BV2 microglia as compared to classical inflammatory activation. Secretion of TNFα increased in a dose-dependent manner in response to LPS treatment (Fig. 2B, ***P < 0.001) but decreased in response to high doses of IL-13 (Fig. 2B, *P < 0.05). Following a scratch wound imposed on a confluent BV2 monolayer, the speed of re-infiltration into the wound was altered by both LPS and IL-13, with divergent effects noted for LPS (which increased infiltration) and IL-13 (which decreased it, Fig. 2C, ***P < 0.001 vs. LPS). Similarly, LPS treatment resulted in altered internal states as indicated by calcium imaging. BV2 cells loaded with Fluo-4 AM displayed a low constitutive frequency of calcium transients that were unaltered by IL-13 but were markedly increased by LPS (Fig. 2D). Finally, we observed changes in the expression of effectors relevant to inflammatory transitions. Mitochondrial enzyme Arginase-1 (Arg-1) was increased following IL-13 treatment (Fig. 2E), while membrane-bound expression of Major Histocompatibility Complex 2 was increased following LPS stimulation (Fig. 2F).

Next, to assess whether these distinct states were associated with transcriptional changes in markers of M1-*like* or M2-*like* activation, we performed RT-qPCR following LPS or IL-13 treatment. The expression of pro-inflammatory markers IL-1β, TNFα, and iNOS were significantly elevated in BV2 cells treated with LPS compared to the untreated control (Fig. 2G–I, *P < 0.05–***P < 0.001). IL-13-treated cells showed no significant difference in expression relative to the untreated control, Conversely, there were significant increases in the expression of the anti-inflammatory markers CD206 (Fig. 2J, *P < 0.05 and Arg-1 (Fig. 2K, ***P < 0.001) following treatment with IL-13. LPS did not induce expression of either marker.

**Fig. 3:**
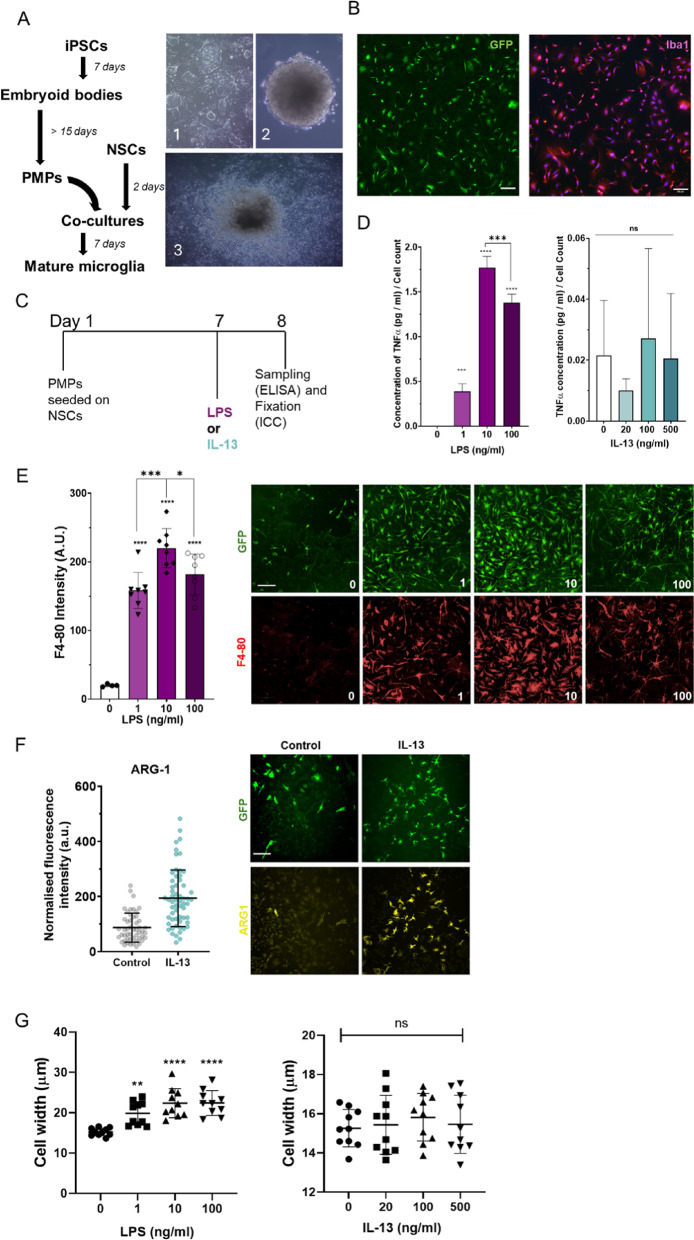
MiPSC-derived microglia respond robustly to both inflammatory and IL-13 signalling.** A** murine iPSCs were cultured for 7 days on a feeder layer of irradiated MEFs [image 1], following this they were developed into embryoid bodies [image 2] that after ~ 15 days in culture regularly released PMPs [image 3]. NSCs were cultured for 2 days before PMPs were added. The NSCs and PMPs were co-cultured together for 7 days to yield GFP expressing mature microglia. **B** The GFP^+^ microglia expressed Iba1. Scale bar, 100 μm. **C** The PMPs were seeded on a layer of NSCs within a 96-well PhenoPlate and incubated for 6 days. On day 7, the cells were treated with LPS (1, 10, or 100 ng/ml) or IL-13 (20, 100, or 500 ng/ml). 24 h later, supernatants were drawn for ELISA, and the cells were fixed with 2% PFA for fluorescence imaging and high-content screening. **D** TNFα secretion was measured using ELISA using supernatant sampled at 24 h after treatment with either LPS or IL-13. The results were normalised to cell count. Data shown as mean ± SD; n = 3–4 biological replicates. **E** F4-80 intensity in cells treated for LPS (1, 10, and 100 ng/ml) for 24 h was quantified using confocal imaging and high-content screening**.** The mean fluorescence intensity of a well of cells was plotted as mean ± SD; n = 4–8 biological replicates with one well taken as an experimental replicate. Scale bar, 50 μm. **F** Confocal imaging and high-content screening of microglia immunostained for Arg-1, these microglia were treated with IL-13 (100 ng/ml) for 24 h. The mean fluorescence intensity of a well of cells was plotted as mean ± SD; n = 50–59 biological replicates with one well taken as an experimental replicate. Scale bar, 50 μm. (**G**) miPSC-microglia increase in width following LPS (1, 10, and 100 ng/ml) treatment in a dose dependent manner while there is no effect observed following IL-13 (20, 100 and 500 ng/ml) treatment. The average cell width per well was plotted as mean ± SD; n = 10 with one well taken as an experimental replicate. One-way ANOVA with Šídák’s multiple comparisons test was applied to panels **D** and **E**. Panel** F** was assessed using an unpaired t-test, statistical analysis for **G** performed using one-way ANOVA with Dunnett’s multiple comparisons test. Unless specified by pairwise annotations, * specifies differences versus control

Finally, we aimed to determine whether miPSC-microglia respond to IL-13 in a similar manner to BV2 cells as miPSC-microglia represent a more analogous model for in vivo microglia. PMPs were generated over a period of ~ 15 days (Figs. 3A, B) prior to co-culture with NSCs. The resulting miPSC-microglia were treated with either LPS or IL-13 (Fig. 3C) at various doses (LPS, at 1, 10, or 100 ng/ml; and IL-13 at 20, 100, or 500 ng/ml). Similarly to data in BV2 microglia, LPS treatment elicited an immune response in the miPSC-microglia with TNFα secretion peaking at LPS 10 ng/ml (Fig. 3D, ****P < 0.0001). Conversely to data in BV2 microglia where IL-13 treatment resulted in a marked decrease in TNFα secretion, there were no changes in TNFα secretion by miPSC-microglia in response to IL-13 at any of the given concentrations (Fig. 3D). There were significant increases in the expression of the glycoprotein F4-80 following LPS treatment with the highest expression quantified at 10 ng/ml dose (Fig. 3E, ****P < 0.0001) compared to untreated cells, while Arg-1 expression increased following IL-13 (100 ng/ml) treatment compared to untreated cells (Fig. 3F). LPS treatment (1, 10, or 100 ng/ml) induced a dose-dependent morphological change in microglia, significantly increasing their width (** *P* < 0.01, ****P < 0.0001)**,** conversely, there were no significant differences in cell width under IL-13 conditions (20, 100, or 500 ng/ml) (Fig. 3G).

Given that IL-13 induced an alternative activation state in both BV2 and miPSC-derived microglia and that this is associated with robust increases in transcription of M2-*like* markers, we reasoned that Arg-1 transcription and/or expression, in particular, would serve as a reliable indicator of effective IL-13 response under different conditions. In this way, we next sought to explore how conditions relevant to neurotrauma might impact IL-13-induced polarisation of microglia.

### Concomitant inflammatory challenge limits IL-13-mediated polarisation of microglia

**Fig. 4:**
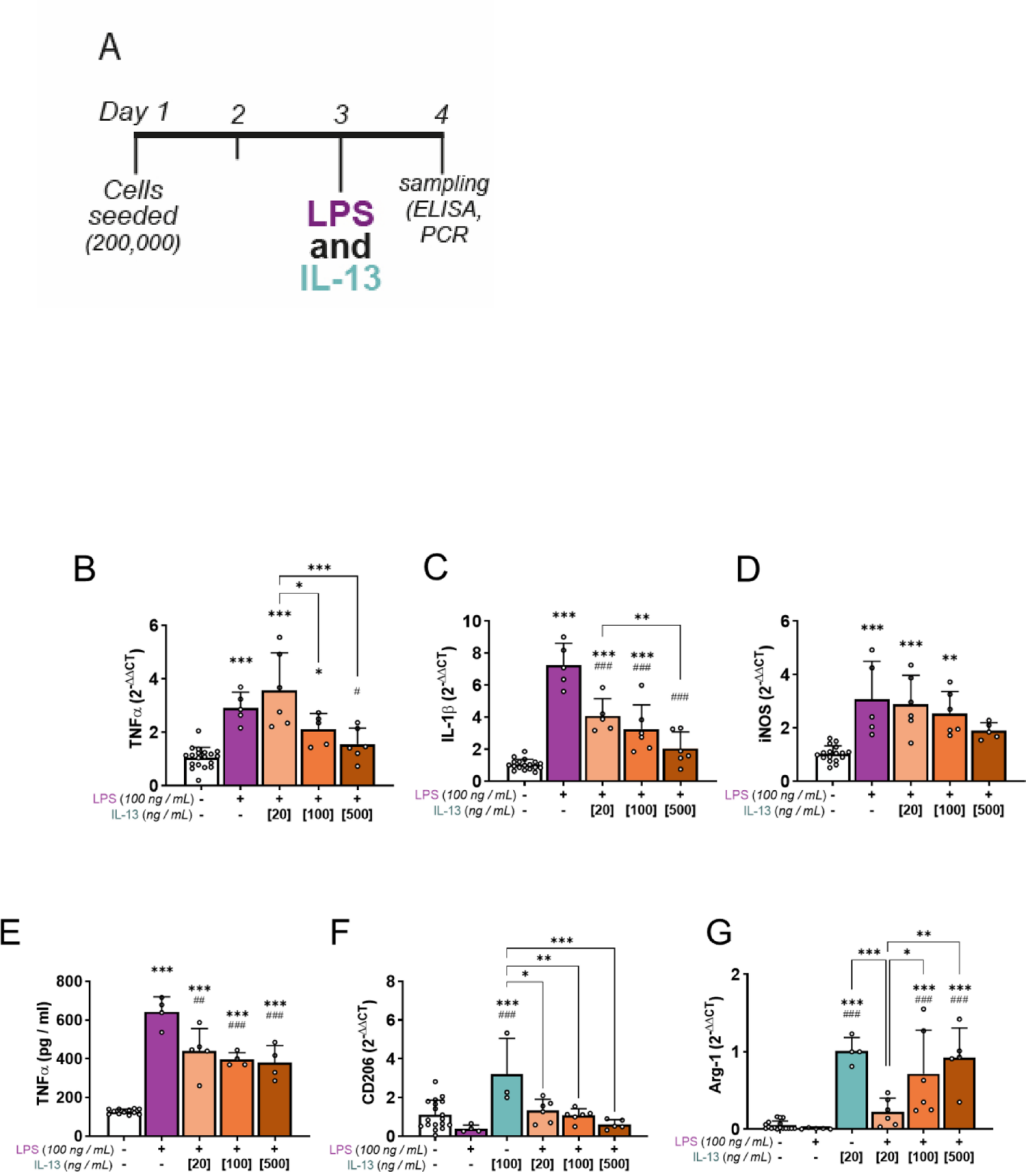
Concomitant inflammatory signals limit forced polarisation by IL-13. **A** To determine whether inflammation limits alternative activation of microglia by IL-13, BV2 microglia were seeded in multi-well culture vessels and incubated for 2 days. On Day 3, cells were treated with LPS (100 ng / mL) and/or IL-13 (20, 100 or 500 ng / mL) prior to sampling for ELISA and RT-qPCR 24 h later. **B-D** Relative transcription of M1-*like* genes TNFα (**B**), IL-1β (**C**) and iNOS (**D**) were measured through quantitative PCR, using PPIA as an endogenous reference gene. Cells were treated with LPS alone or in combination with increasing doses of IL-13. **E** TNFα secretion was measured using ELISA, with supernatant sampled at 24 h after treatment. **F-G** Relative transcription of M2-*like* genes CD206 (**F**) and Arg-1 (**G**) were measured through quantitative PCR, as above in **B**-**D**. Data shown as mean ± SD; n = 4–18 biological replicates, sampled from at least three independent culture expansions. All panels involving quantitative data (**B–G**) were assessed using one-way ANOVA with Šídák’s multiple comparisons test. Unless specified by pairwise annotations, * specifies differences versus control, while **#** denotes differences versus LPS alone

To explore whether IL-13 maintains its efficacy in polarising microglia during inflammatory conditions, BV2 cells were treated concomitantly with both LPS (100 ng/ml) and various doses of IL-13 (20, 100 or 500 ng/ml, Fig. 4A). As before, we assessed transcription of pro-inflammatory markers TNFα, IL-1β, and iNOS, as well as the anti-inflammatory markers CD206 and Arg-1, using RT-qPCR. LPS alone induced significant increases in expression of TNFα, IL-1β, and iNOS, as expected (Fig. 4B–D). However, co-treatment with IL-13 yielded mixed results and failed to reduce expression of pro-inflammatory genes below control levels. TNFα expression, for instance, remained significantly elevated following LPS, despite co-treatment with IL-13 (20 ng/mL and 100 ng/mL, Fig. 4B, *P < 0.05–***P < 0.001); while at very high doses of IL-13 (500 ng/mL), expression remained 48% higher than control levels. Similarly, IL-1β expression was significantly higher than control levels following co-treatment with LPS and IL-13 (20 ng/mL and 100 ng/mL, Fig. 4C, ***P < 0.001). Although co-treatment with IL-13 (20 ng/mL and 100 ng/mL) reduced the levels of IL-1β transcription when compared to LPS only, it remained elevated compared to control samples, and transcription of IL-1β was 97% higher than control samples following co-treatment with high doses of IL-13 (500 ng/mL). Finally, LPS-induced expression of iNOS was unaltered following co-treatment with all doses of IL-13 (Fig. 4D). We performed ELISA to confirm whether TNFα secretion is decreased following co-treatment with LPS and IL-13. Although IL-13 co-treatment reduced the levels of extracellular TNFα, it remained significantly elevated compared to controls (Fig. 4E, ***P < 0.001).

We then followed a similar approach to assess anti-inflammatory markers following co-treatment with LPS and IL-13. IL-13 failed to induce CD206 transcription when paired with LPS challenge (Fig. 4F, *P < 0.05–***P < 0.001 for all doses of IL-13 paired with LPS). Meanwhile, IL-13-mediated induction of Arg-1 expression was significantly diminished during co-treatment with LPS (20 ng/mL IL-13, Fig. 4G, ***P < 0.001). Although IL-13 remained effective in inducing Arg-1 expression, it required far higher doses of IL-13 to match the effects seen without LPS co-treatment. These results indicate that concomitant pro-inflammatory signals, such as those acting via Toll-like receptors, limit the capacity of IL-13-induced polarisation.

### Extrinsic factors associated with neurotrauma constrain microglial phenotype and limit forced polarisation by IL-13

**Fig. 5:**
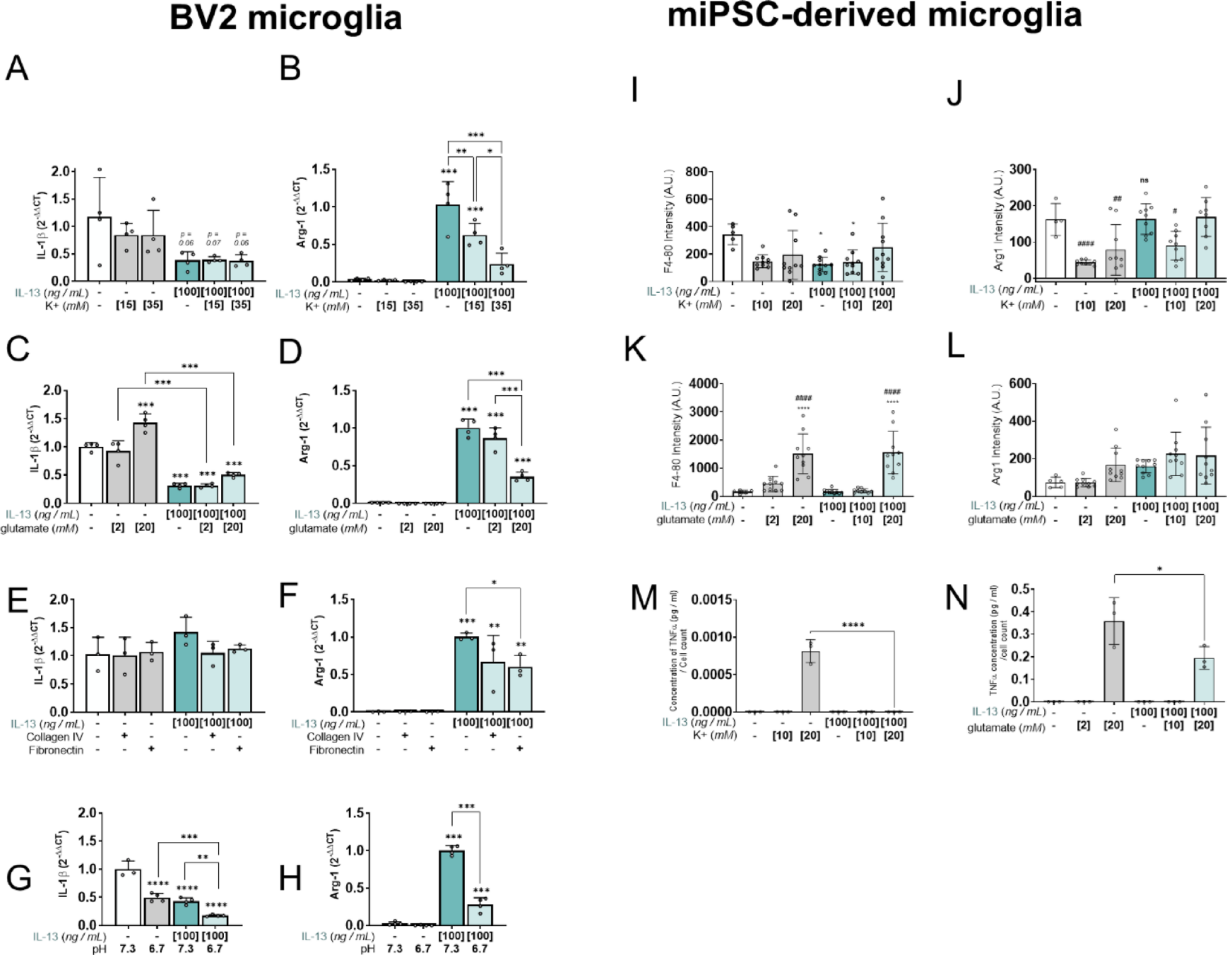
Extrinsic factors associated with neurotrauma limit forced polarisation by IL-13 in BV2 and miPSC-microglia. To simulate the effects of microenvironmental disruption seen during neurotrauma and whether this interferes with forced polarisation by IL-13, BV2 microglia were treated with IL-13 (100 ng/mL) in a variety of culture conditions prior to lysis and RNA sampling for RT-qPCR, 24 h after treatment with IL-13. Murine iPSC-microglia were treated with IL-13 (100 ng/mL) and either KCl or glutamate prior to sampling for ELISA and fixation for high-content screening. **A**, **B** BV2 cells were grown for 48 h before treatment with IL-13 (100 ng/mL) or low-serum media control. To induce hyperkalemia, treatments were given in supplemented media with a final [K^+^]_e_, 15 mM or 35 mM. **C**, **D** BV2 cells were grown for 48 h before treatment with IL-13 (100 ng/mL) or low-serum media control. To induce hyperglutamate, treatments were given in supplemented media with a final [glutamate]_e_ of 2 mM or 20 mM. **E**, **F** BV2 cells were grown in multi-well plates coated with collagen IV (10 μg/ml) or fibronectin (10 μg/ml) for 48 h before treatment with IL-13 (100 ng/mL) or low-serum media control. **G**, **H** BV2 cells were grown for 48 h before treatment with IL-13 (100 ng/mL) or low-serum media control. To assess the polarising ability of IL-13 on microglia under acidic conditions, media used for treatments was adjusted from a pH of 7.3 to a pH of 6.7 sterile filtered before use. Relative transcription of M1-*like* gene IL-1β (**A, C, E, G**) and M2-*like* gene Arg-1 (**B, D, F, H**) were measured through RT-qPCR, using PPIA as an endogenous reference gene. **I**, **J** miPSC-microglia were cultured for 6 days before treatment with IL-13 (100 ng/ml). To induce hyperkalaemia, treatments were given in supplemented media with a final [K^+^]_e_, of 10 mM or 20 mM. **K**,** L** miPSC-microglia were cultured for 6 days before treatment with IL-13 (100 ng/ml). To induce hyperglutamate, treatments were given in supplemented media with a final [glutamate]e of 2 mM or 20 mM. **M**, **N** TNFα secretion by miPSC-microglia under hyperkalaemia or hyperglutamate conditions was quantified and normalised to cell count. Expression of M1-*like* marker F4-80 (**I**, **K**) and M2-*like* marker Arg-1 (**J**, **L**) were quantified using confocal imaging and high-content screening. Data obtained from BV2 microglia shown as mean ± SD; n = 3–4 biological replicates, sampled from at least 3 independent cultures. Data obtained from miPSC-microglia shown as mean intensity per well in a single data point, mean ± SD; n = 3–10 biological replicates. All statistical analyses performed using one-way ANOVA with Šídák’s multiple comparisons test. Unless specified by pairwise annotations, * denotes significant differences versus control and * denotes differences versus IL-13 alone

Neurotrauma leads to profound changes in the extracellular microenvironment, with potential implications on the success of forced polarisation of microglia through IL-13 treatment. Here, we sought to alter key culture conditions to resemble those seen following traumatic injury. Lesions associated with neurotrauma exhibit maladaptive conditions such as hyperkalemia, hyperglutamate and acidosis, so we next sought to address whether such conditions limit IL-13 effects on microglia. BV2 cells were cultured in hyperkalemic media (15 mM and 35 mM [K^+^]_e_), and transcription of IL-1β and Arg-1 were measured in response to IL-13. IL-1β expression was unaltered in hyperkalemic conditions; however, there was a dose-dependent decrease in Arg-1 induction following treatment with IL-13 in hyperkalemic media, compared to IL-13 treatment in isokalemic media (Fig. 5A and B, **P < 0.01, ***P < 0.001). Similarly, BV2 microglia grown in hyperglutamate culture media (2 mM and 20 mM [glu]e) showed limited IL-13-mediated Arg-1 induction at high glutamate tone (Fig. 5D, ***P < 0.001). In this case, however, this was accompanied by significant decreases in IL-1β expression following IL-13 treatment (Fig. 5C, ***P < 0.001). This effect was evident even under high glutamate load, indicating that IL-13-mediated polarisation is altered but not abolished, leading to alternative microglial phenotypes that do not exhibit M2-*like* changes in Arg-1 expression. Additionally, we tested whether increased deposition of extracellular matrix (ECM) proteins altered IL-13 polarisation in BV2 microglia. Culture dishes were coated with either collagen IV (10 μg/ml) or fibronectin (10 μg/ml) and transcription of IL-1β and Arg-1 was measured, as previously, in response to IL-13. IL-1β expression was not altered in the presence of either collagen IV or fibronectin (Fig. 5E). However, although IL-13 induced significant increases in Arg-1 transcription in all conditions, IL-13-induced transcription of Arg-1 was significantly lower in microglia grown on fibronectin substrate (Fig. 5F, *P < 0.05). Finally, in BV2 microglia, we assayed IL-13-mediated activation in acidic media (pH 6.7). As seen during hyperglutamate conditions, microglia response to IL-13 was altered in acidic media. IL-13 treatment resulted in decreased IL-1β transcription (Fig. 5G) and increased Arg-1 transcription (Fig. 5H). However, concomitant acidosis was associated with lower IL-1β expression and lower Arg-1 transcription when compared to IL-13 treatment in control media, signifying a quiescent phenotype. In miPSC-microglia, F4-80 and Arg-1 expression were quantified, using high-content screening, under hyperkalemic or hyperglutamate conditions in response to IL-13. Both F4-80 and Arg-1 expression were unaltered in hyperkalemic conditions (10 mM and 20 mM [K^+^]_e_) (Fig. 5I and J). Following treatment of miPSC-microglia with 2 mM or 20 mM [glu]e, there was significant increase in F4-80 expression at the 20 mM concentration which was not attenuated by the addition of IL-13 (Fig. 5K). There were no changes in Arg-1 expression under hyperglutamate conditions (Fig. 5L). Following concomitant IL-13 and 20 mM [K^+^]_e_ treatment of miPSC-microglia, there was a significant decrease in TNFα secretion compared to 20 mM [K^+^]_e_ treatment (Fig. 5M). Similarly, the addition of IL-13 to 20 mM [glu]e treated cells significantly reduced TNFα secretion compared to 20 mM [glu]e alone. Taken together, these data suggest that microenvironmental contexts associated with neurotrauma alter microglial response to IL-13, limiting the extent of M2-*like* expression and pushing microglia into more quiescent states.

### LPS priming entrains mixed phenotypes in microglia following IL-13 treatment

**Fig. 6:**
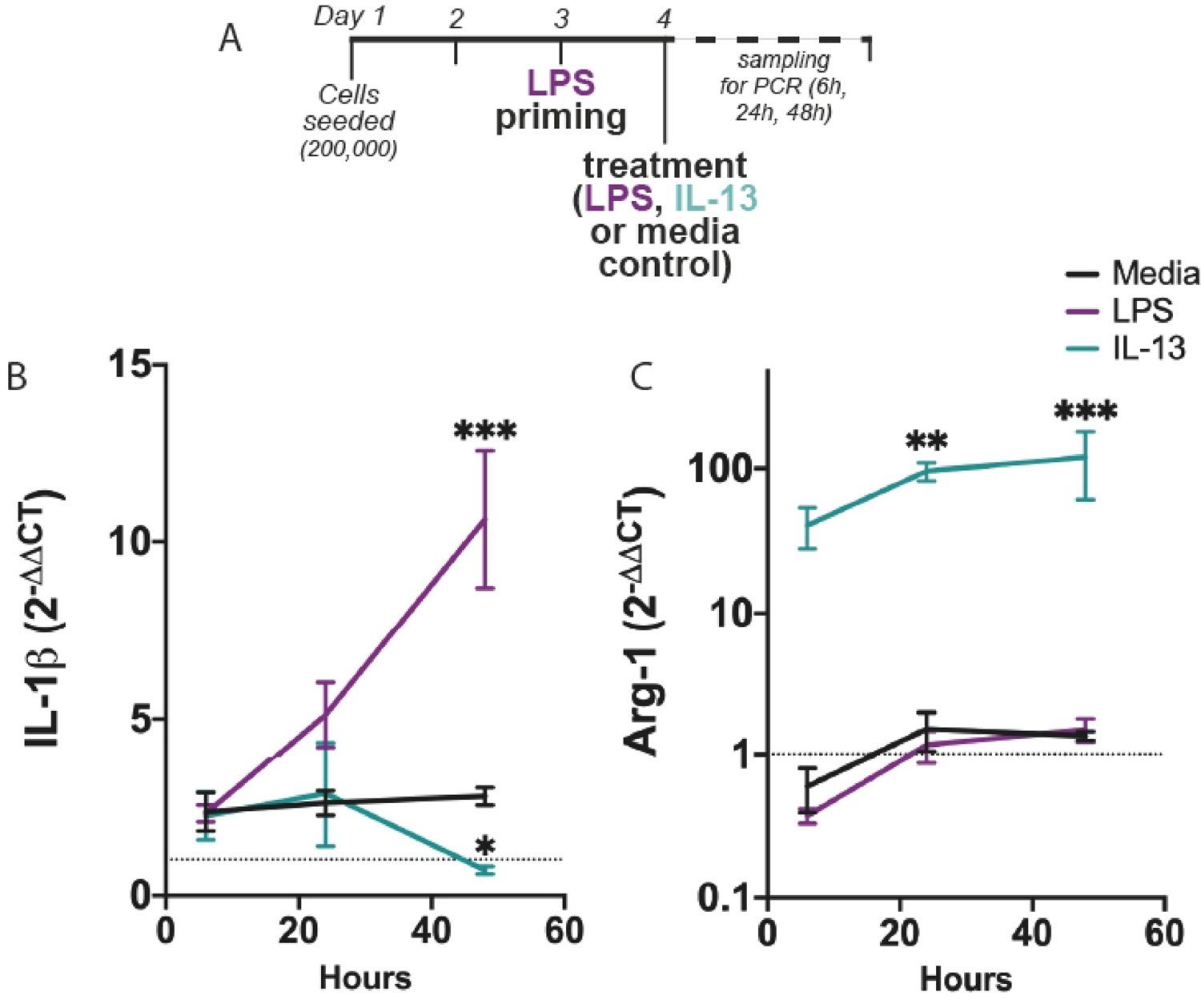
Inflammatory priming entrains mixed phenotypes in microglia following IL-13 treatment.** A** To determine if prior inflammatory signalling entrains persistent effects that limit alternative activation by IL-13, BV2 microglia were treated with LPS (24 h priming, 100 ng / ml) prior to treatment with either LPS (100 ng/mL), IL-13 (100 ng/mL) or low-serum media control. Cells were then lysed and processed for RNA isolation and RT-qPCR. **B** BV2 cells were primed and subsequently treated, before IL-1β transcription was assessed at 6, 24 and 48 h following treatment. Relative RNA levels were quantified and normalised to non-primed control (*dashed line*), using PPIA as an endogenous reference gene. **C** Arg-1 transcription in BV2 microglia was assessed following priming and treatment, as in **B**. Data shown as mean ± SEM; n = 3–10 biological replicates, sampled from at least 3 independent cultures. All statistical analysis performed using two-way ANOVA with Benjamini, Krieger and Yekutieli False Discovery Rate post-hoc test. * denotes significant differences versus time-matched primed control

Inflammation may confer residual effects on microglia that persist after cessation of inflammatory signals. Here, we primed BV2 microglia with LPS (100 ng/mL) for 24 h, before treating cells with either LPS (100 ng/mL), IL-13 (100 ng/mL) or low-serum media control (Fig. 6A), to determine if inflammatory priming limits forced polarisation of microglia. We assayed transcription of both IL-1β and Arg-1 at 6, 24 and 48 h following treatment, relative to a non-primed control.

LPS priming invoked an elevated level of IL-1β transcription that persisted for at least 48 h (media control), although the effect size was substantially less than seen after persistent inflammatory challenge with LPS for 48 h (Fig. 6B, ***P < 0.001). Although IL-13 reduced IL-1β transcription, it took 48 h to do so, with no apparent reduction at 6 or 24 h (Fig. 6B, *P < 0.05). In contrast, IL-13 readily induced Arg-1 transcription within 24 h, even after LPS priming (Fig. 6C, **P < 0.01). These data suggest that inflammatory priming of microglia entrains mixed phenotypes that exhibit transcription of both M1-*like* and M2-*like* for limited periods during IL-13 forcing.

### Recapitulation of microglial developmental cues facilitates forced polarisation by IL-13 during inflammation.

Development and maintenance of homeostatic microglia in the CNS is dependent upon a variety of growth factors and modulation signals. Previous data has identified that activation of TGFβ receptors and CSF-1 receptors is necessary, both to drive microglial lineage commitment during development and to maintain microglial homeostatic identity in the CNS [25, 30, 31]. We reasoned that recapitulating these signalling cascades might restore normative states in microglia that render them more amenable to IL-13 forcing during inflammation. To this end, we supplemented culture medium with TGFβ1, TGFβ2, M-CSF, and cholesterol in parallel to treating microglia with both IL-13 and LPS. Further, we also sought to develop a high-content screening method that could be leveraged to rapidly test other signalling factors that may facilitate better polarisation by IL-13 into alternatively activated states.

**Fig. 7:**
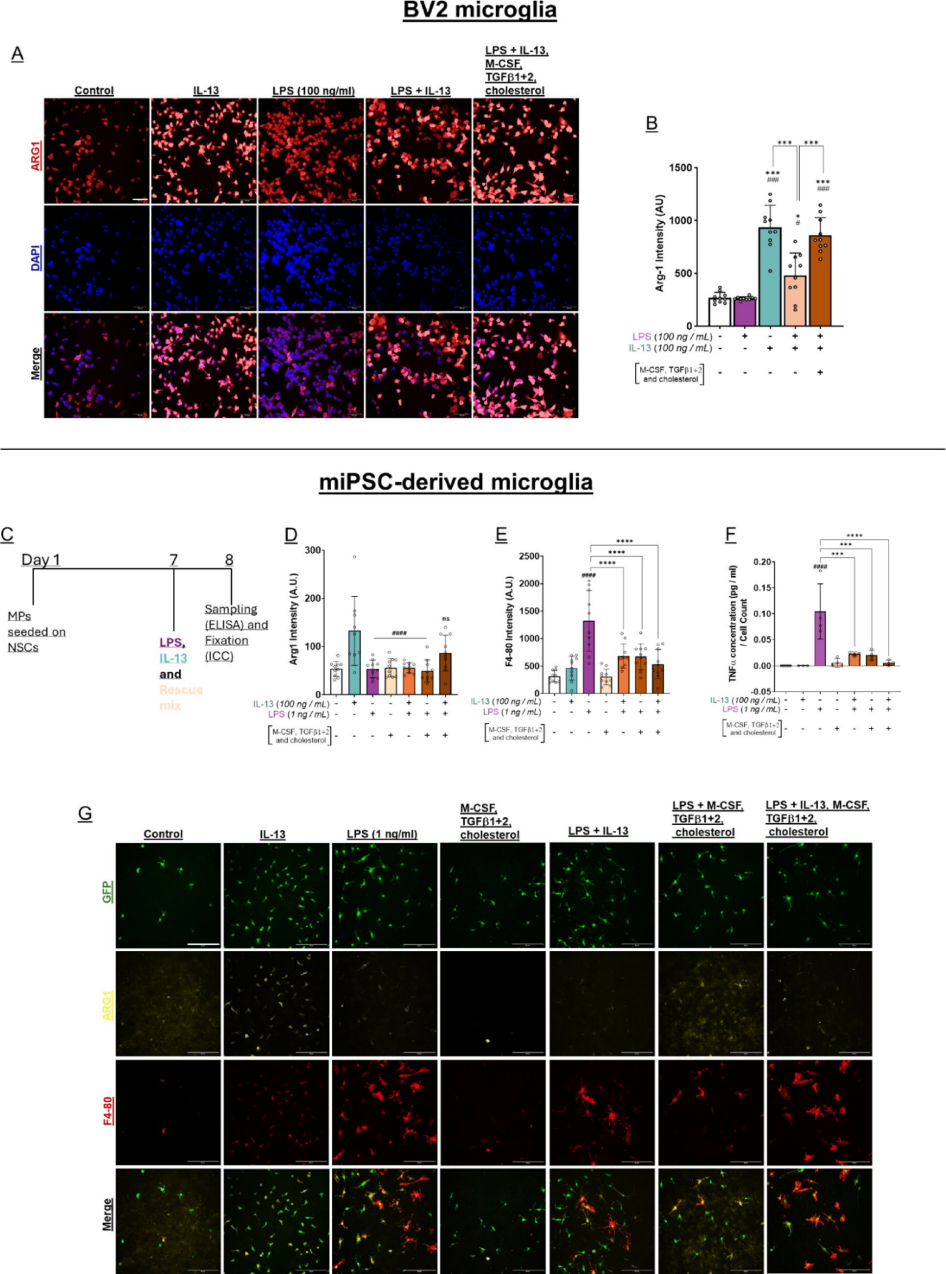
Rescuing homeostatic signals unlock forced polarisation of microglia by IL-13 during inflammation. High-throughput confocal microscopy was employed to rapidly determine if recapitulating developmental lineage cues could improve forced polarisation by IL-13 during classical inflammation. **A** BV2 cells were cultured in 96-well optical plates and treated with combinations of LPS (100 ng/ml), IL-13 (100 ng/mL), M-CSF, TGFβ1, TGFβ2 and/or cholesterol (1.5 μg/mL). Cells were fixed and processed for immunostaining and high-throughput fluorescence microscopy. Scale bar, 100 μm. **B** Quantification of high-throughput confocal microscopy data from **A**. Hoescht-positive cells were segmented using CellMask and Arg-1 immunostaining intensity was quantified per cell. Aggregate cell data is shown, with mean ± SD; n = 10 sampled from a single BV2 passage. *P < 0.05, ***P < 0.001; one-way ANOVA with Šídák’s multiple comparisons test. Unless specified by pairwise annotations, * specifies differences versus control, while **#** denotes differences versus LPS alone. **C** PMPs were cultured on a layer of NSCs within 96-well optical plates and after 7 days in culture, were treated with combinations of LPS (1 ng/ml), IL-13 (100 ng/ml), M-CSF (10 ng/ml), TGFβ1 (22 mg/ml), TGFβ2 (5 mg/ml), and/or cholesterol (1.5 mg/ml). After 24 h, samples were taken for ELISA, cells were fixed and processed for immunostaining and high-throughput confocal microscopy. **D** Arg-1 intensity was quantified in GFP + cells only. Mean intensity per well is shown as a single data point, mean ± SD; n = 10 biological replicates from two separate experiments. **E** F4-80 intensity was quantified in GFP + cells only. Mean intensity per well is shown as a single data point, mean ± SD; n = 10 biological replicates from two separate experiments. **F** TNFα secretion was measured using ELISA, 24 h after treatment, mean ± SD; n = 4 biological replicates from two separate experiments. **G** Images were taken at 20X magnification. Scale bar, 50 μm. All statistical analyses performed using one-way ANOVA with Šídák’s multiple comparisons test. Unless specified by pairwise annotations, * denotes significant differences versus control and **#** denotes differences versus IL-13 alone

BV2 cells were cultured in 96-well optical plates, and the efficacy of IL-13 polarisation was assessed via expression of Arg-1 protein, as measured through immunofluorescence and high-throughput confocal microscopy (Fig. 7A). Arg-1 expression was low in control and LPS-treated cells. IL-13 treatment led to a robust increase in Arg-1 expression, as expected; however, concomitant treatment with LPS and IL-13 limited Arg-1 induction (Fig. 7B, ***P < 0.001), as seen previously (see Fig. 4G) The addition of TGFβ1, TGFβ2, M-CSF, and cholesterol to the culture media led to a robust rescue of Arg-1 induction when microglia were treated with both LPS and IL-13, yielding levels of Arg-1 expression similar to that seen in cells treated with IL-13 only (Fig. 7B, ***P < 0.001). Microglia progenitors were cultured in 96-well optical plates on a layer of NSCs to promote microglial maturation. The effect of IL-13 on mature miPSC-microglia polarisation was quantified via Arg-1 and F4-80 expression using high-content screening, and TNFα secretion was measured using ELISA (Fig. 7C). Arg-1 expression was low in control-, LPS-, TGFβ1, TGFβ2, M-CSF-, and cholesterol-treated groups, while treatment with IL-13 resulted in a significant increase in Arg-1 expression (Fig. 7D), while concomitant treatment with LPS and IL-13 limited Arg-1 induction in these microglia (Fig. 7D ####*P* < 0.0001). Similar to data in BV2 microglia, addition of TGFβ1, TGFβ2, M-CSF, and cholesterol to LPS and IL-13-treated miPSC-microglia, led to an increase in Arg-1 induction resulting in levels of Arg-1 expression similar to that seen in cells treated with IL-13 only. Conversely to data in BV2 microglia, treatment with TGFβ1, TGFβ2, M-CSF, cholesterol, LPS and IL-13 group was not significantly different from the concomitantly treated LPS and IL-13 group in miPSC-microglia.

There was a significant decrease in F4-80 expression in LPS-treated groups supplemented with IL-13 and/or TGFβ1, TGFβ2, M-CSF, and cholesterol in comparison to the LPS-only group (Fig. 7E), suggesting that these homeostatic factors are capable of dampening the inflammatory effect without the addition of IL-13. MiPSC-microglia treated with LPS in combination with IL-13 and/or TGFβ1, TGFβ2, M-CSF, and cholesterol showed significant decreases in TNFα secretion compared to LPS treated cells (Fig. 6F). Expression of Arg-1 and F4-80 were measured and visualized using high-throughput confocal microscopy (Fig. 7G).

These data indicate that recapitulating homeostatic cues facilitates IL-13 polarisation of microglia into M2-*like* states. Such signalling cascades can overcome persistent pro-inflammatory signals, suggesting that forced polarisation may be a viable strategy in neurotrauma, but only if additional forcing agents are supplemented. These data also demonstrate the applicability of optical screening in rapidly identifying other candidate factors that can boost IL-13 efficacy.

## Discussion

IL-13 has been shown to polarise microglia towards an alternatively activated anti-inflammatory phenotype categorized by release of neurotrophic factors and tissue repair [31]. Although IL-13 delivery into the spinal cord improved functional and histopathological recovery after SCI in murine models [5], this was primarily driven by macrophage polarisation into pro-reparative M2-*like* phenotypes, with limited effects mediated by microglia. Microglia respond robustly to IL-13 in vitro, suggesting that concomitant factors arising in vivo following neurotrauma limit the IL-13 response in microglia. Shortly following an SCI, microglia predominantly shift towards a classically activated phenotype characterised by phagocytosis and release of pro-inflammatory cytokines [10]. However, the broad population exhibits phenotypic diversity related to the extent and severity of injury that evolves with time [16]. We hypothesized that a number of extrinsic factors associated with neurotrauma constrain microglial phenotype and reduce the efficacy of IL-13 to force M2-*like* phenotypes. If distinct patterns of activation arise in situ, these factors may even preclude immunomodulation where it would be most beneficial. We demonstrated in vitro that extrinsic factors including pro-inflammatory signals, ECM deposition, acidosis, excessive glutamate release and hyperkalemia all contribute to this effect, reducing the efficacy of IL-13 in forcing M2-*like* polarisation in BV2 and miPSC-microglia. These data suggest that extrinsic factors arising following neurotrauma can limit immunomodulatory strategies and that multimodal modulation may be necessary to force anti-inflammatory and neuroregenerative properties in microglia.

In this study, we utilised two stable, complementary microglial models: immortalised BV2 cells, valued for their reproducibility and homogeneity [32, 33] and miPSC derived microglia, which retain the capacity to acquire CNS specific cues [34].

Notably, BV2 microglia are less sensitive to LPS in comparison to miPSC-microglia as they lack some specific genes and epigenetic regulators [35, 36]. Here, we confirmed that IL-13 has distinct polarising effects on both BV2 and miPSC-derived microglia in comparison to pro-inflammatory stimulants. LPS induces classical inflammation in microglia through Toll-like receptor 4 activation and downstream induction of NFkB, MAP kinases and interferon-regulatory factors, among other signalling cascades, leading to the release of pro-inflammatory cytokines [37–40]. LPS is widely used to induce acute inflammation in a wide variety of studies including microglia studies [41]. We found that LPS triggered increased secretion and expression of pro-inflammatory markers IL-1β, TNFα, and iNOS in BV2 microglia, and increased TNFα secretion and expression of the activation marker F4-80 in miPSC-microglia. These effects were in contrast to the alternative phenotype induced by IL-13, indicated by the differential expression of the anti-inflammatory markers CD206 in BV2 cells and Arg-1 in both BV2 and miPSC-microglia models. Supported by the literature [35, 36], data presented here demonstrates that miPSC-microglia are more sensitive to LPS stimulation in comparison to BV2 microglia with TNFα secretion, and F4-80 expression peaking at 10 ng/ml (Fig. 3D, E), whereas TNFα secretion in BV2 microglia peaked at 1 μg/ml LPS (Fig. 2). Additionally, morphological changes were observed in miPSC-microglia (Fig. 3G), with cell width significantly increasing under LPS stimulation versus no changes under IL-13 treatment. Morphological changes are an indicator of microglia activation [42] and as demonstrated here, these changes are occurring under inflammatory conditions reinforcing their utility as a marker of immune responsiveness.

Other cellular behaviours, including scratch-wound response and intracellular calcium activity, were also distinct, confirming that IL-13 leads to alternative activation of BV2 microglia. Alternative activation of microglia may be a promising avenue to induce neuroregenerative and anti-inflammatory processes in the CNS. IL-13 immune gene therapy induced alternative activation in microglia in vivo confirmed via the expression of Arg-1 [43]. IL-13 treatment also reduced TNFα expression in microglia within cuprizone demyelination models. This microglial phenotype was associated with increased oligodendrocyte survival and reduced demyelination, where IL-13 increased the phagocytic activity of microglia resulting in clearance of myelin debris, and reductions in the level of neuroinflammation and demyelination. Additionally, IL-13 activity on microglia can improve neurofunctional recovery following ischemic stroke [44], enhance anti-inflammatory crosstalk between astrocytes and microglia in Alzheimer’s disease [45] and promote long-term functional recovery following traumatic brain injury by enhancing microglia phagocytic activity and dampening pro-inflammatory responses [46].

Following SCI, there is a rapid loss of microglia at the lesion site and the remaining microglia release damage-associated molecular patterns (DAMPs) that trigger chronic neuroinflammation [10, 13]. A key DAMP that is released by microglia following SCI is HMGB1 (high-mobility group box-1). The extracellular concentration of HMGB1 increases due to secretion by microglia and necrotic neurons and this exacerbates inflammation via recruitment of immune cells and further cytokine release [47]. Extracellular HMGB1 activates inflammatory cells predominantly via the RAGE, TLR2, and TLR4 pathways. This persistent inflammation could be an important factor restricting IL-13 polarisation. Here, treatment of microglia with LPS during or prior to IL-13 activation, reduces the extent of alternative activation. IL-13 did not effectively reduce expression of pro-inflammatory genes including TNFα, IL-1β, and iNOS in BV2 microglia, or the activation marker F4-80 in miPSC-microglia when paired with TLR4 activation by LPS. This occlusion is also seen in primary microglia, where IL-13 only partially reduces IL-1β transcription when cells are concomitantly treated with LPS and IFNγ [9]. Similarly, the IL-13-mediated induction of anti-inflammatory markers CD206 and Arg-1 was occluded in the presence of pro-inflammatory challenge. Furthermore, we observed that transient LPS stimulation can entrain microglia for periods as long as 48 h, conferring residual phenotypes defined by continued release of pro-inflammatory factors, despite IL-13-mediated induction of Arg-1. LPS priming is known to limit microglia activation [48]. IL-13 was unable to reduce neuroinflammation 6 h following TBI and was reported to be less effective at promoting M2-*like* microglia phenotypes [46]. Here too, LPS priming may have rendered the microglia less able to switch to M2-*like* phenotypes in response to IL-13, resulting in mixed populations or phenotypes.

We also examined the effects of ECM proteins, given the high degree of release and deposition of ECM proteins after SCI, including cleaved hyaluronan, tenascin-C, fibronectin and other fragmented proteoglycans [49]. We found that fibronectin hinders IL-13’s efficacy as shown by the lowered Arg-1 expression in IL-13 treated microglia seeded on fibronectin. Fibronectin is a component of the fibrous scar that forms around, and within, the SCI lesion, and is primarily secreted by reactive astrocytes. Through binding with β1 integrin receptor, fibronectin can polarise microglia to a pro-inflammatory phenotype [50], and trigger increased expression of MHC class I and upregulation of alpha-integrins [51]. Furthermore, expression of matrix metalloproteinase 9 (MMP9) by microglia is upregulated by fibronectin [52]. MMP9 in large quantities is linked to blood–brain barrier disruption, particularly through delayed damage by degrading tight junction proteins such as occludin and claudin [53] and components of the basement membrane, including collagen IV [54]. Additionally, MMP9 contributes to the activation of pro-inflammatory cytokines such as TNFα, thus propagating chronic inflammation [55]. MMP9 is activated through the NF-kB/AP1 pathway [56]. Although IL-13 suppresses TNFα-induced activation of NF-kB/AP1, over-production of inflammatory cytokines could lead to receptor saturation whereby all IL-13Ra are fully bound thus limiting further IL-13 activity [57, 58]. Thus, the effect of fibronectin on microglial activation and subsequent expression of MMP9 may be a key factor in hindering IL-13 action on microglia.

SCI can also trigger excessive accumulation of extracellular glutamate and K^+^. Traumatic impact causes mechanical disruption to the primarily astrocyte-mediated transporter systems that maintain glutamate and K + homeostasis. In normal physiological conditions, extracellular K^+^ is maintained at around 3 mM [59, 60]. The potassium transporter Kir4.1 expressed by astrocytes functions to buffer extracellular K^+^ accumulation [61]. After an SCI, chronic loss of Kir4.1 expression, particularly at the lesion centre [62], may subsequently lead to higher extracellular K^+^, neuronal cell death via excitotoxicity and spreading depolarisation that can transiently increase [K^+^]_e_ as high as 60 mM [62–64]. High extracellular K^+^ has been reported to disrupt astrocytic Na^+^/K^+^/ATPase activity, further contributing to the homeostatic imbalance after SCI [65, 66]. Moreover, increased extracellular K^+^ can activate microglia, inducing TNFα release [67] and neurotoxic phenotypes [68]. The reduction in IL-13 efficacy during [K^+^]_e_ supplementation seen here in BV2 cells indicates that microglia are responsive to hyperkalemia. Raising [K^+^]_e_ precluded Arg-1 induction in a dose-dependent manner, hindering IL-13 polarising efficacy. Similarly, in miPSC-microglia, [K^+^]_e_ treatment led to significant decreases in Arg-1 expression compared to IL-13 treatment. Alongside the elevation of [K^+^]_e_, the extracellular concentration of glutamate is upregulated concomitantly, and the inflammatory cascade closely follows [69].

Estimates of extracellular glutamate concentrations within the CNS vary widely depending on region and technique used. Estimates drawn from electrophysiological and/or calcium imaging approaches report steady-state levels in the nanomolar range (30—50 nM in hippocampus [70], and 25—150 nM in the nucleus accumbens [71], for example, while in vivo microdialysis tends to indicate levels within micromolar range [72]. This includes spinal cord where glutamate increases ten-fold following SCI, nearing millimolar range [73]. Although such discrepancies may be due to compromised glutamate reuptake at the probe arising from glial scarring, excessive glutamate release following SCI is certain. During inflammation, reactive astrocytes exhibit reduced re-uptake through GLAST (EAAT1) and GLT-1 (EAAT2) [74], while synaptic vesicles harbouring as much as 200 mM glutamate can escape and rupture during neuronal cell death [75], thus contributing to increased extracellular glutamate. To mimic this glutamate accumulation, microglia cells were exposed to excessive glutamate concentrations, accounting for high tolerance of glutamate in otherwise healthy CNS cells [76, 77]. The addition of IL-13 decreased glutamate-induced IL-1β expression in BV2 microglia adding credence to the studies suggesting that glutamate has a role in inflammation, but again, IL-13 efficacy on Arg-1 induction was limited by glutamate in a dose-dependent matter. Moreover, the addition of IL-13 was unable to decrease glutamate-induced F4-80 expression in miPSC-microglia. While there was a significant decrease in TNFα secretion in miPSC-microglia following treatment with IL-13, this effect was limited by high glutamate concentrations. Under neuroinflammatory states, microglia express the glutamate transporters GLT-1 and GLAST, as well as AMPA and Kainate receptors, and mGluR2, 3 and 5 [78]. The binding of glutamate to the AMPA or Kainate receptors stimulates microglial production of TNFα [79]. Furthermore, when glutamate binds to mGluR2, there is additional release of glutamate triggered by the release of TNFα, and production of nitric oxide [80]. This receptor binding may be a contributing factor to the decreased Arg-1 expression we show here, where excessive glutamate may trigger feed-forward processes that sustain chronic inflammation and free radical release by microglia.

Extracellular pH is typically around pH 7.3. Tissue acidosis (pH < 7.0) is a common occurrence following traumatic injury to the CNS and is associated with more pronounced motor deficits during the secondary injury phase of SCI [81]. Excess extracellular glutamate leads to acidosis via excitotoxicity, and inflammation can also induce local acidosis [82]. There have been various channels implicated in mediating trauma-associated acidosis including the acid-sensing ion channels (ASICs) and the voltage-gated proton channel Hv1. ASIC1a expression and activity is upregulated following SCI, particularly in spinal neurons [82]. Hv1 is selectively expressed by microglia and was also found to be involved in pathological acidosis following SCI [80]. Our data confirms that acidosis at this level hinders IL-13 efficacy leading to poor induction of Arg-1 following IL-13 treatment. Hv1 may be the ion channel involved in the limiting of IL-13 activity. Hv1 deficiency in ischemic mice led to significant increases in CD206 and Arg-1 expression, while there was a decrease seen in iNOS and CD16 expression compared to the wildtype mice [83]. This is an indication that Hv1 may be involved in inflammatory microglia polarisation potentially through production of reactive oxygen species. Additionally, IL-4 was used to force M2-*like* polarisation in microglia obtained from wildtype mice and Hv1 deficient mice. Again, the expressions of CD206 and Arg-1 were upregulated in Hv1 deficient microglia compared to wildtype mice in the presence of IL-4. Like IL-13, IL-4 acts as an anti-inflammatory polarising force on microglia and both IL-4 and IL-13 interact through a common receptor, heterodimer of IL-4Rα and IL-13Rα1 [19, 30, 44, 81]. Thus, Hv1 activity may be similarly inhibiting IL-13 function.

The findings of this study suggest that IL-13 has distinct polarising effects on microglia, promoting an anti-inflammatory M2-*like* phenotype while not inducing a strong proinflammatory M1-*like* phenotype like LPS. This suggests that forced polarisation of microglia towards an anti-inflammatory phenotype is a possible immunomodulatory tactic to be considered. Nevertheless, here we present a myriad of factors that affect IL-13 efficacy within the cellular microenvironment, which highlights a limitation that can arise with a unimodal treatment strategy. Based on this we then sought to determine the potential of a multimodal approach by adding factors that may act synergistically with IL-13 and boost forced polarisation of microglia. TGFβ1, TGFβ2, and M-CSF play crucial roles in microglia development. Mice deficient for TGFβ1 in the brain or spinal cord have defects in extracellular glutamate homeostasis [84]. TGFβ1 is a major differentiation factor for microglia, and the lack thereof is associated with microglial depletion and multifocal autoinflammatory disorder [84]. Other studies found that TGFβ1 treated macrophages had higher expression of M2-*like* markers such as IL-10 and Arg-1 compared to non-treated cells [85]. Other studies have discussed the enhancing effects of TGFβ1 on IL-4 induced microglia alternative activation [86]. Similar anti-inflammatory activities have been demonstrated by TGFβ2 [87]. M-CSF promotes the development and maintenance of microglia and can also polarise macrophages towards an M2-*like* phenotype. Using Multiplex ELISA, it was determined that M-CSF is an ‘M2’ polarising factor [88]. While cholesterol is not known to induce anti-inflammatory polarisation in microglia, it is a key component of cell membranes and cellular structures. A reduction in cholesterol biosynthesis during chronic neuroinflammatory responses affects microglia activation capabilities and normal metabolic activities. Cholesterol signalling alongside TGFβ, and M-CSF is important for maintaining homeostatic microglia conditions and regulating neuroinflammatory responses [89, 90]. Here we show that a combination of TGFβ1, TGFβ2, M-CSF, and cholesterol, can boost IL-13 polarising activity as shown by the increase in Arg-1 expression in BV2 and miPSC-microglia, even in the presence of significant LPS challenge. We adapted this treatment to a high-content screening approach, validating a method to rapidly screen for agents that improve the efficacy of IL-13 in forced polarisation of microglia. Given the heightened sensitivity of miPSC-microglia to LPS, we selected a lower concentration of 1 ng/mL for these experiments. In contrast, BV2 microglia responded to higher concentrations of LPS more robustly (100 ng/ml), showing a marked increase in Arg-1 expression upon IL-13 (100 ng/ml) stimulation and further enhancement in the presence of TGFβ1, TGFβ2, M-CSF, and cholesterol signalling. TGFβ1, TGFβ2, M-CSF, and cholesterol signalling maintained effectiveness in miPSC-microglia co-treated with IL-13 and LPS. Arg-1 expression in cells treated with the full combination of LPS, IL-13, TGFβ1, TGFβ2, M-CSF, and cholesterol reached levels similar to those induced by IL-13 alone. Although Arg-1 levels did not significantly differ between the IL-13 + LPS group and the full combination group (Fig. 7) these data suggests that this strategy offers a potentially effective approach to modulate miPSC-microglial activation toward an anti-inflammatory, Arg-1-expressing state. Moreover, this promising strategy can be readily applied within other relevant conditions including hyperkalaemia, excitotoxicity, matrix protein deposition, and acidosis, and within more sophisticated co-cultures. These results contribute to a better understanding of the molecular mechanisms underlying microglial polarisation and suggest a path forward for the development of multimodal approaches that better unlock phenotypic forcing in microglia.

## Data Availability

Datasets generated and/or analysed during this study are available from the corresponding author upon reasonable request.
